# Control of Long-Term Plasticity by Glutamate Transporters

**DOI:** 10.3389/fnsyn.2019.00010

**Published:** 2019-04-09

**Authors:** Silvana Valtcheva, Laurent Venance

**Affiliations:** Dynamics and Pathophysiology of Neuronal Networks Team, Center for Interdisciplinary Research in Biology (CIRB), Collège de France, CNRS UMR7241/INSERM U1050, Paris, France

**Keywords:** synaptic plasiticty, excitatory amino acid transporters, glutamate uptake, astrocytes, spike-timing dependent plasticity, neuro-glia crosstalk, glutamate spillover

## Abstract

Activity-dependent long-term changes in synaptic strength constitute key elements for learning and memory formation. Long-term plasticity can be induced *in vivo* and *ex vivo* by various physiologically relevant activity patterns. Depending on their temporal statistics, such patterns can induce long-lasting changes in the synaptic weight by potentiating or depressing synaptic transmission. At excitatory synapses, glutamate uptake operated by excitatory amino acid transporters (EAATs) has a critical role in regulating the strength and the extent of receptor activation by afferent activity. EAATs tightly control synaptic transmission and glutamate spillover. EAATs activity can, therefore, determine the polarity and magnitude of long-term plasticity by regulating the spatiotemporal profile of the glutamate transients and thus, the glutamate access to pre- and postsynaptic receptors. Here, we summarize compelling evidence that EAATs regulate various forms of long-term synaptic plasticity and the consequences of such regulation for behavioral output. We speculate that experience-dependent plasticity of EAATs levels can determine the sensitivity of synapses to frequency- or time-dependent plasticity paradigms. We propose that EAATs contribute to the gating of relevant inputs eligible to induce long-term plasticity and thereby select the operating learning rules that match the physiological function of the synapse adapted to the behavioral context.

## Introduction

**Figure 1:**
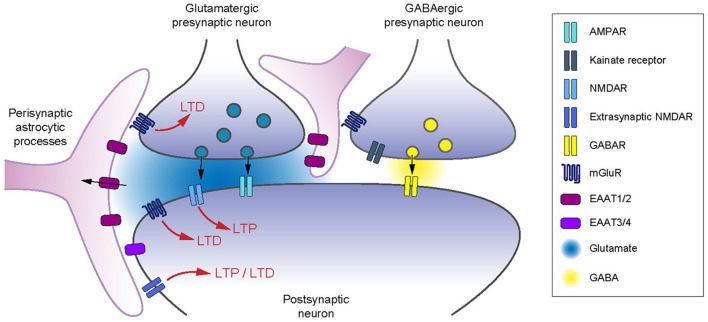
Excitatory amino acid transporters (EAATs) control of receptors involved in long-term synaptic plasticity. EAATs control the activation of pre- and postsynaptic glutamate receptors, as well as the spread of glutamate to neighboring inhibitory neurons. EAAT2 controls the induction of long-term plasticity relying on presynaptic (Omrani et al., 2009) and postsynaptic mGluRs (Brasnjo and Otis, 2001; Valtcheva and Venance, 2016) and postsynaptic NMDARs (Katagiri et al., 2001; Massey et al., 2004; Wong et al., 2007; Scimemi et al., 2009; Valtcheva and Venance, 2016). Astrocytic coverage of neurons controls the activation of presynaptic type-III mGluRs or kainate receptors on GABAergic terminals (Piet et al., 2004; Bonfardin et al., 2010).

Information processing at central synapses is governed by two main neural coding strategies: integration and coincidence detection, which rely on the rate- and spike-time coding, respectively (deCharms and Zador, 2000; Brette, 2015). Accordingly, the ability of synapses to undergo long-term changes in synaptic weight have been investigated *in vivo* and *ex vivo* using two main types of cell conditioning paradigms: rate-based and spike-timing-based protocols (Malenka and Bear, 2004; Sjöström et al., 2008; Feldman, 2012). The induction of long-term potentiation (LTP) or depression (LTD), following different cell conditioning paradigms, is assessed by the relative change in the magnitude of postsynaptic responses. The induction of long-term synaptic plasticity at glutamatergic synapses requires the activation of presynaptic and postsynaptic glutamate receptors, situated at synaptic, perisynaptic and extrasynaptic sites (Asztely et al., 1997; Bergles and Jahr, 1997; Bergles et al., 1997; Min et al., 1998; Rusakov and Kullmann, 1998; Lehre and Rusakov, 2002; Zheng et al., 2008; Figure 1). The timing of activation of glutamate receptors is expected to be proportional to their distance from the presynaptic release site (Attwell and Gibb, 2005). There is a critical role of glutamate diffusion in determining the balance of receptor activation. High-affinity membrane glutamate transporters (also named excitatory amino acid transporters, EAATs) control the degree to which glutamate receptors located in the perisynaptic space or outside the synaptic cleft are activated following each release event (Bergles et al., 1997; Min et al., 1998; Zheng et al., 2008; Vandenberg and Ryan, 2013). The glutamate uptake process is electrogenic and is driven by the ion gradients of K^+^ and Na^+^ (Zerangue and Kavanaugh, 1996; Levy et al., 1998; Owe et al., 2006). EAATs have similar affinities for glutamate as glutamate receptors (Arriza et al., 1994) and their transport cycle is slow relative to the time course of glutamate in the synaptic cleft (Clements et al., 1992; Wadiche et al., 1995; Bergles and Jahr, 1998). Therefore, the main role of EAATs is to terminate the glutamate transient by primary acting as glutamate buffers followed by active transport. While during sparse activation of synapses, glutamate is likely cleared from the synaptic cleft by diffusion instead of active transport (Helassa et al., 2018), EAATs appear as key players for plasticity induction by controlling the spatiotemporal activation of glutamatergic receptors during episodes of high neuronal activity.

EAATs are constituted by five subtypes, named EAAT1-5 (Danbolt, 2001). EAAT type-1 (EAAT1) and type-2 (EAAT2) are mainly expressed in glial cells. EAAT1 is mostly expressed by Bergmann glia cells but is also found in other brain regions (Arriza et al., 1994; Rothstein et al., 1994; Chaudhry et al., 1995; Lehre et al., 1995; Wadiche and Kavanaugh, 1998). EAAT2 is specifically expressed in perisynaptic astrocytic processes ensheathing synaptic complexes, but not in astrocytic cell bodies (Danbolt et al., 1992; Rothstein et al., 1994; Lehre et al., 1995; Furuta et al., 1997; Levy et al., 1998; Minelli et al., 2001; Holmseth et al., 2009). EAAT2 can also be found in some excitatory neurons in hippocampus and cortex but its physiological role remains uncertain based on its distribution (not concentrated at synapses) and its low level of expression (~10% of astrocytic EAAT2; Chen et al., 2004; Furness et al., 2008; Melone et al., 2009, 2011; Petr et al., 2015; Danbolt et al., 2016; Rimmele and Rosenberg, 2016). EAAT type-3 (EAAT3) and EAAT type-4 (EAAT4) are found in neurons at postsynaptic sites (Rothstein et al., 1994; Fairman et al., 1995; Lehre et al., 1995; Furuta et al., 1997; Conti et al., 1998). EAAT4 is expressed by cerebellar Purkinje cells in particular on extrasynaptic sites (Tanaka et al., 1997; Dehnes et al., 1998). Finally, EAAT type-5 (EAAT5) is expressed in the photoreceptors, bipolar and amacrine cells of the retina and has been suggested to mainly act as glutamate-activated chloride channel to control the excitability of retinal neurons (Eliasof and Jahr, 1996; Arriza et al., 1997; Veruki et al., 2006; Schneider et al., 2014).

Numerous studies have shown that both astrocytic and neuronal EAATs regulate the output of activity-dependent long-term synaptic plasticity triggered by different cell conditioning paradigms. Pharmacological or genetic alteration of EAATs activity can either facilitate or impair rate-based synaptic plasticity (Brasnjo and Otis, 2001; Katagiri et al., 2001; Massey et al., 2004; Wang et al., 2006; Wong et al., 2007; Omrani et al., 2009; Bellini et al., 2018). Spike-timing-based cell-conditioning paradigms such as spike-timing-dependent plasticity (STDP) reveal subtler multidimensional regulation of synaptic plasticity by EAATs. STDP is a Hebbian synaptic learning rule accounting for experience-dependent changes in neural networks (Sjöström et al., 2008; Feldman, 2012), which depends on pre- and post-synaptic activity. Besides the change in synaptic weight, STDP rules are described by the permissive temporal window (Δt_STDP_) for plasticity expression. If pre- and post-synaptic activity occur at Δt_STDP_ beyond a few tens of milliseconds, it does not generally trigger long-term synaptic efficacy changes, and, therefore, these events are considered as uncorrelated. Altering glutamate uptake by up- or downregulation of the astrocytic EAAT2 has an effect on both STDP expression and the temporal range of its permissive window (Valtcheva and Venance, 2016).

Here, we review recent findings on the control of long-term synaptic plasticity by EAATs and we discuss how alterations of glutamate transport affect behavior. Finally, we focus on the physiological regulation of EAATs by different forms of experience and speculate on how this might shape the sensitivity of synapses to undergo different forms of long-term plasticity.

## EAATs Control of Long-Term Synaptic Plasticity Depends on the Cell Conditioning Paradigm

EAATs exert differential control on long-term synaptic plasticity depending on the cell conditioning paradigm, i.e., rate- vs. spike-timing protocols. EAATs regulate the expression and magnitude of rate-based synaptic plasticity, whereas EAATs control the expression and the temporal window of spike-timing-based synaptic plasticity.

**Figure 2:**
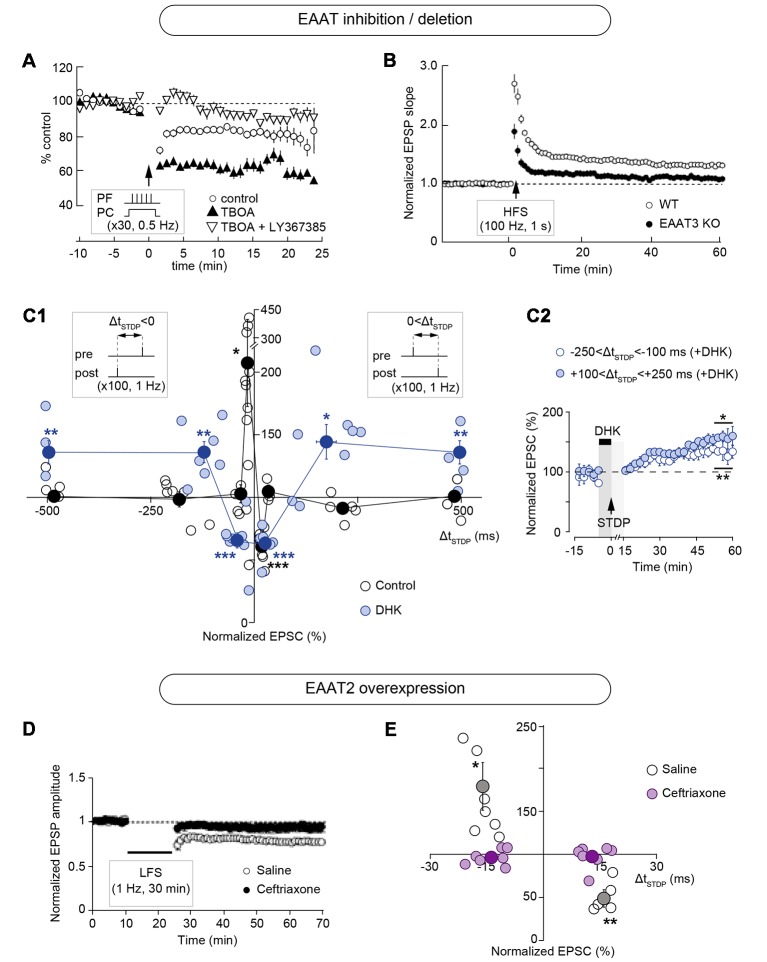
Modulation of long-term synaptic plasticity by EAATs. **(A)** Pharmacological inhibition of EAATs by the broad-spectrum non-transportable blocker threo-β-benzyloxyaspartic acid (TBOA) increases the magnitude of long-term depression (LTD) in cerebellar Purkinje cells, triggered by high frequency stimulation (HFS) of the parallel fibers (five pulses at 100 Hz) paired with Purkinje cell depolarization (0 mV for 50 ms) repeated at 0.5 Hz for 1 min. This LTD is mGluR1-dependent since it is inhibited by LY367385. Adapted with permission from Brasnjo and Otis (2001). **(B)** Genetic deletion of EAAT2 decreases the amplitude of HFS-induced long-term potentiation (LTP) in the stratum radiatum region of the hippocampus. Adapted with permission from Katagiri et al. (2001). **(C)** Specific pharmacological blockade of EAAT2 with dihydrokainic acid (DHK) results in a broadening of the permissive window for spike-timing dependent plasticity (STDP) expression at corticostriatal synapses. Inhibition of EAAT2 results in t-LTD or t-LTP at temporal intervals where no plasticity is observed under control conditions (beyond Δt_STDP_ = ±30 ms). **(C1)** Time window (Δt_STDP_) for long-term synaptic strength for post-pre and pre-post pairings. **(C2)** Averaged time-course of experiments with transient blockade of EAAT2 with bath-application of DHK for post-pre and pre-post pairings beyond Δt_STDP_ = ±100 ms. Adapted with permission from Valtcheva and Venance (2016). **(D)** Overexpression of EAAT2 by chronic ceftriaxone treatment prevents the expression of low frequency stimulation (LFS)-induced LTD at the hippocampal mossy fibers-CA3 synapse. Adapted with permission from Omrani et al. (2009). **(E)** STDP time window (Δt_STDP_) for long-term synaptic strength for post-pre and pre-post pairings showing that overexpression of EAAT2 by chronic ceftriaxone treatment prevents the expression of STDP at corticostriatal synapses. Adapted with permission from Valtcheva and Venance (2016). *p < 0.05; **p < 0.01; ***p < 0.001.

**Figure 3:**
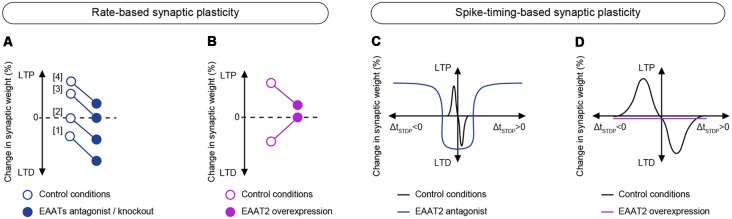
Effects of EAATs modulation on long-term plasticity. **(A,B)** Effects of EAATs regulation on rate-based plasticity. **(A)** Downregulation of EAATs by pharmacological inhibition or genetic knock-out (closed circles) results in a lower threshold for LTD induction and a higher threshold for LTP induction using rate-based plasticity paradigms compared to control conditions (open circles). Pharmacological inhibition of EAATs: (1) increases LTD magnitude (Brasnjo and Otis, 2001); (2) promotes LTD (Massey et al., 2004; Wong et al., 2007); (3) prevents LTP induction (Wang et al., 2006). Genetic knock-out of EAATs; and (4) decreases LTP amplitude (Katagiri et al., 2001; Scimemi et al., 2009). **(B)** Upregulation of EAATs by chronic ceftriaxone treatment (closed circles) prevents the expression of LFS-induced LTD and decreases the amplitude of HFS-induced LTP compared to control conditions (open circles; Omrani et al., 2009). **(C,D)** Effects of EAATs regulation on spike-timing-based plasticity. **(C)** Downregulation of EAAT2 by pharmacological inhibition with DHK results in a broadening of the permissive window (Δt_STDP_) for plasticity expression (Valtcheva and Venance, 2016). **(D)** Upregulation of EAATs by chronic ceftriaxone treatment prevents the expression of STDP (Valtcheva and Venance, 2016).

Pharmacological or genetic targeting of EAATs alters the magnitude of plasticity induced with rate-based protocols such as low- and high-frequency stimulation (LFS and HFS, respectively) or theta-burst stimulation (TBS; Figures 2, 3; Table 1). Inhibition of glutamate uptake with bath application of threo-β-benzyloxyaspartic acid (TBOA), a broad-spectrum non-transportable EAATs blocker, or with *trans*-4-carboxy-L-proline (t-PDC), a transportable inhibitor, promotes LFS-LTD in layer II/III of perirhinal cortex of adult rats while no plasticity is observed in control conditions (Massey et al., 2004; Figure 3A). Similarly, TBOA bath application in acute brain slices or intracerebroventricular infusion *in vivo* also promotes LFS-LTD in the CA1 region of the hippocampus of adult rats, while no plasticity is observed after LFS in control conditions *in vivo* and *ex vivo* (Wong et al., 2007; Figure 3A). Blockade of glutamate transport with TBOA also increases single-cell LTD magnitude in cerebellar Purkinje cells in juvenile rats, triggered by HFS of the parallel fibers paired with Purkinje cell depolarization (Brasnjo and Otis, 2001; Figures 2A, 3A). Genetic deletion of astrocytic EAAT2 results in impaired HFS-LTP, but has no effect on LFS-LTD, in brain slices of the stratum radiatum of mice (Katagiri et al., 2001; Figures 2B, 3A). Pharmacological blockade with the EAAT2 specific inhibitor dihydrokainic acid (DHK) decreases TBS-LTP magnitude of C-fiber evoked field potentials in the spinal dorsal horn of anesthetized rats (Wang et al., 2006; Figure 3A). TBS-LTP in the hippocampal CA1 region is impaired in mice lacking neuronal EAAT3 (Scimemi et al., 2009; Figure 3A). Overexpression of EAAT2 protein levels *via* i.p. treatment with the beta-lactam antibiotic ceftriaxone (Rothstein et al., 2005) prevents the expression of LFS-LTD and decreases the magnitude of HFS-LTP at the hippocampal mossy fibers-CA3 synapse (Omrani et al., 2009; Figures 2D, 3B).

**Table 1:** Effect of excitatory amino acid transporters (EAATs) down- or up-regulation on rate-based and spike-timing-based synaptic plasticity.

Reference	Structure/synapse	Manipulation/drug	Recording	Brain slice cutting conditions	Plasticity paradigm	Effect on plasticity
**Downregulation**
Brasnjo and Otis (2001)	Cerebellum, parallel fiber-Purkinje neurons	TBOA, bath-application	*Ex vivo*, whole-cell, EPSCs	Ice-cold (4°C) ACSF	HFS + postsynaptic depolarization	Increases LTD magnitude
Katagiri et al. (2001)	Hippocampus stratum radiatum	Knock-out	*Ex vivo*, extracellular, fEPSPs	ice-cold (4°C) ACSF	HFS and LFS	Decreases HFS-LTP magnitude; no effect on LFS-LTD
Massey et al. (2004)	Perirhinal cortex, layer II/III pyramidal neurons	TBOA, bath-application	*Ex vivo*, extracellular, fEPSPs	Ice-cold (4°C) ACSF	HFS and LFS	Promotes LFS-LTD induction
Tsvetkov et al. (2004)	Cortex-/Thalamus-Lateral amygdala pyramidal neurons	DHK, bath-application	*Ex vivo*, whole-cell, EPSCs	*	LFS	Promotes heterosynaptic LFS-LTP plasticity of unpaired input
Wang et al. (2006)	C-fibers-spinal dorsal horn	DHK, *in vivo* intrathecal administration	*In vivo*, extracellular, fEPSPs	N/A	HFS	Decreases HFS-LTP amplitude
Wong et al. (2007)	Hippocampus Schaffer collateral-CA1	TBOA, *ex vivo* bath- application or *in vivo* intraventricular infusion	*Ex vivo* and *in vivo*, extracellular, fEPSPs	Ice-cold (4°C) ACSF	HFS and LFS	Promotes LFS-LTD induction
Scimemi et al. (2009)	Hippocampus CA1	knock-out	*Ex vivo*, extracellular, fEPSPs	Ice-cold (4°C) ACSF	TBS and LFS	Impaires TBS-LTP
Valtcheva and Venance (2016)	Somatosensory cortex- dorsolateral striatum output neurons	DHK or WAY-213,613, bath-application	*Ex vivo*, whole-cell, EPSCs	Ice-cold (4°C) ACSF	STDP	Induces non-Hebbian LTP Induces heterosynaptic LTD
**Overexpression**
Omrani et al. (2009)	Hippocampus Mossy fiber- CA3 Hippocampus Schaffer collateral-CA1	Ceftriaxone, i.p. injection	*Ex vivo*, extracellular, fEPSPs	Ice-cold (4°C) ACSF	HFS and LFS	MF-CA3: impairs LFS-LTD and decreases magnitude of HFS-LTP Schaffer collateral-CA1: no effecton HFS-LTP magnitude
Valtcheva and Venance (2016)	Somatosensory cortex- dorsolateral striatum output neurons	Ceftriaxone, i.p. injection	*Ex vivo*, whole-cell, EPSCs	Ice-cold (4°C) ACSF	STDP	Occludes t-LTP and t-LTD

*Not mentioned in the Methods; N/A, not applicable.

Exploring STDP expression with specific pharmacological targeting of EAAT2 glutamate uptake uncovers that together with its control over plasticity magnitude, EAAT2 tightly regulates the temporal window for plasticity induction in the rat dorsolateral striatum (Valtcheva and Venance, 2016; Figures 2C, 3C; Table 1). Indeed, specific pharmacological blockade of EAAT2 (with DHK or WAY-213,613) during the STDP induction protocol results in a broadening of the permissive window for plasticity expression at corticostriatal synapses. Inhibition of EAAT2 promotes the expression of spike-timing-dependent LTD (t-LTD) or spike-timing-dependent LTP (t-LTP) at temporal intervals, where no plasticity is observed under control conditions (beyond Δt_STDP_ = ±30 ms for 1 Hz pairings). Upon EAAT2 inhibition, plasticity expression does not follow STDP rule, as a non-timing-dependent LTP can be triggered by uncorrelated events (randomized pre-post and post-pre pairings) or even unpaired activity (postsynaptic activation alone). EAAT2 inhibition reveals an overlap between non-timing-dependent LTP and LTD triggered by the recruitment of GABAergic networks at a narrow temporal window (−70 < Δt_STDP_ < +70 ms; Valtcheva and Venance, 2016). On the contrary, overexpression of EAAT2 following chronic ceftriaxone treatment of rats results in a lack of STDP at corticostriatal synapses (Valtcheva and Venance, 2016; Figures 2E, 3D). Therefore, EAAT2 control the expression of spike-timing-based plasticity but also acts as a selector for Hebbian vs. non-Hebbian plasticity.

## How EAATs Control Long-Term Plasticity

EAATs regulate the expression of both rate- and spike-timing-based long-term plasticity by several mechanisms. EAATs control the extent of activation of receptors present in synaptic and peri-/extrasynaptic compartments, or on neighboring neurons, by controlling the temporal and spatial profile of the glutamate transient (Tong and Jahr, 1994; Brasnjo and Otis, 2001; Clark and Cull-Candy, 2002; Dzubay and Otis, 2002; Reichelt and Knöpfel, 2002; Attwell and Gibb, 2005; Tzingounis and Wadiche, 2007; Chalifoux and Carter, 2011). Synaptically released glutamate diffuses out of the synaptic cleft and binds to NMDARs and mGluRs in the peri- or extrasynaptic membrane or at neighboring synapses (Kullmann et al., 1996; Barbour and Häusser, 1997; Scanziani et al., 1997; Kullmann and Asztely, 1998; Szapiro and Barbour, 2007). The extent of such extrasynaptic actions is regulated by the high-affinity glutamate uptake operated mainly by EAAT2 (Asztely et al., 1997; Bergles and Jahr, 1997; Min et al., 1998; Rusakov and Kullmann, 1998; Lehre and Rusakov, 2002; Zheng et al., 2008). EAAT2 rapidly reduces the free concentration of glutamate but part of the content of the exocytosis of presynaptic vesicles binds to glutamatergic receptors situated in the immediate perisynaptic space (Rusakov and Kullmann, 1998; Zheng et al., 2008). Moreover, sustained episodes of high-frequency presynaptic activity delay glutamate clearance by astrocytes and allow prolonged activation of NMDARs (Armbruster et al., 2016). Finally, diffusion of EAAT2 on the astrocytic membrane is crucial for the glutamate buffering function of EAAT2, thus allowing the transporter to relocate between synaptic and peri-/extrasynaptic sites (Murphy-Royal et al., 2015). There is a critical role of glutamate diffusion in determining the balance of receptor activation and EAATs control the degree to which receptors located outside the cleft are activated following each release event (Bergles et al., 1997; Min et al., 1998; Zheng et al., 2008).

### EAATs Control the Activation of Receptors Involved in Long-Term Plasticity

Up- or down-regulation of glutamate uptake can have a profound effect on plasticity expression or magnitude. Increase in EAATs density would increase the buffering capacity for glutamate and decrease glutamate receptor stimulation as EAATs compete for the extracellular glutamate with NMDARs and mGluRs located in the peri- or extrasynaptic space (Bergles et al., 2002; Tzingounis and Wadiche, 2007; Figure 1). Increasing glutamate clearance can also reduce synaptic receptor activation (Min et al., 1998). In contrast, downregulation of EAATs should allow extended binding of glutamate to its receptors (Figure 1).

Up-regulation of EAAT2, with ceftriaxone (Rothstein et al., 2005), prevents LFS-LTD and decreases the magnitude of HFS-LTP at the hippocampal mossy fibers-CA3 synapse (Omrani et al., 2009; Figures 2D, 3C). The effect on LFS-LTD can be explained by the enhanced glutamate clearance resulting from EAAT2 up-regulation, thus limiting the activation of perisynaptic mGluRs which are responsible for LFS-LTD induction at the mossy fibers-CA3 synapse (Kobayashi et al., 1996; Yokoi et al., 1996). Indeed, bath-applied DHK during LFS rescues LTD in ceftriaxone-treated animals, by promoting glutamate accumulation and mGluRs activation (Omrani et al., 2009). Likewise, reduced glutamate access to presynaptic kainate receptors, which mediates HFS-LTP at the mossy the fibers-CA3 synapse (Schmitz et al., 2003) might account for the reduction in HFS-LTP magnitude. A similar mechanism is likely involved in the loss of striatal t-LTD and t-LTP expression after EAAT2 up-regulation with ceftriaxone treatment (Valtcheva and Venance, 2016; Figures 2E, 3D). At corticostriatal synapses in the dorsolateral striatum, t-LTD is mGluR-mediated and t-LTP depends on NMDARs (Fino et al., 2010; Evans et al., 2012), and these receptors are located within but also outside the synaptic cleft (Baude et al., 1993; Paoletti et al., 2013). Therefore, EAAT2 overexpression may limit the activation of receptors responsible for STDP induction and impair the detection of correlated synaptic activity.

EAATs inhibition by TBOA increases the magnitude of associative mGluR-dependent LTD at cerebellar parallel fiber-Purkinje cell synapse by enhancing mGluRs transmission (Brasnjo and Otis, 2001; Figures 2A, 3A). Specific pharmacological blockade of EAAT2 with intrathecal infusion of DHK in the spinal dorsal horn decreases HFS-LTP magnitude at lower doses of DHK and precludes LTP expression at higher doses (Wang et al., 2006; Figure 3A). LTP of unmyelinated C-fibers onto spinal dorsal horn neurons synapse relies on NMDARs activation (Randić et al., 1993). Therefore, continuous EAAT2 blockade (*via* intrathecal DHK infusion) and subsequent glutamate accumulation may result in excessive NMDAR stimulation, neuronal depolarization and subsequent increase in intracellular Ca^2+^ concentration, which would hinder further LTP induction. Similarly, genetic deletion of either astrocytic EAAT2 (Katagiri et al., 2001) or neuronal EAAT3 (Scimemi et al., 2009) impairs HFS-LTP in the hippocampus, most likely due to the chronic activation of NMDARs (Figure 3A). In knock-out mice for either EAAT2 or EAAT3 (Katagiri et al., 2001), glutamatergic transmission is increased, which may lead to enhanced stimulation of NMDARs, therefore precluding the ability of synapses to further potentiate.

Distinct natural expression patterns of EAATs also affect plasticity expression in different regions. Indeed, Purkinje cells in the cerebellar vermis express lower levels of EAAT4 and exhibit mGluR-dependent LFS-LTD at the parallel fiber-Purkinje cell synapse. In contrast, LFS-LTD is absent in the cerebellar flocculus where EAAT4 is highly expressed, which might decrease the extent of mGluRs recruitment during HFS (Wadiche and Jahr, 2005).

### EAATs Prevent the Expression of Aberrant Plasticity

EAATs control the extent of glutamate spillover and the access of glutamate to receptors located at the peri- and extrasynaptic sites (Figure 1). Although EAATs are not overwhelmed by physiological activity, a total synaptic isolation is not reached (Asztely et al., 1997; Diamond and Jahr, 1997, 2000). Receptors that are typically not recruited by glutamate release under afferent stimulation in control conditions, can become key actors of long-term plasticity when glutamate uptake is altered (Tzingounis and Wadiche, 2007). Therefore, physiological or pathological down-regulation of EAATs has a permissive role for the expression of different forms of long-term plasticity (Massey et al., 2004; Wong et al., 2007; Aida et al., 2015; Valtcheva and Venance, 2016). EAATs prevent the expression of aberrant plasticity that is absent in physiological conditions but can be revealed when EAATs are blocked. Both rate- (Massey et al., 2004; Wong et al., 2007) and spike-timing-based (Valtcheva and Venance, 2016) paradigms which fail to induce changes in synaptic strength in control conditions *in vivo* and *ex vivo*, can promote long-term plasticity when glutamate uptake is impaired.

Pharmacological inhibition of all EAAT subtypes by TBOA in the perirhinal cortex (Massey et al., 2004) and in the CA1 region of the hippocampus (Wong et al., 2007) has a permissive role for the expression of *in vivo* and *ex vivo* LFS-LTD, mediated by NMDARs containing the GluN2B-subunit (Figure 3A). In the dorsolateral striatum, specific EAAT2 inhibition by DHK (or WAY-213,613) disrupts the temporal contingency between pre- and postsynaptic activity, which is required for STDP expression (Valtcheva and Venance, 2016; Figures 2C, 3C). The sharp sensitivity to the timing of synaptic inputs of striatal STDP is erased in conditions of EAAT2 blockade when DHK (or WAY-213,613) is applied exclusively during the STDP protocol. This promotes the expression of a non-timing-dependent LTP mediated by GluN2B-NMDARs which is can also be induced by uncorrelated pre- and postsynaptic events. This non-timing-dependent LTP is absent in control conditions when EAAT2 activity is intact.

### EAATs Ensure Synaptic Independence

Astrocytic EAAT2 is of crucial importance for limiting glutamate spillover to neighboring synapses, and therefore tightly controlling both cooperation and synaptic independence (Arnth-Jensen et al., 2002; Huang et al., 2004; Scimemi et al., 2004; Attwell and Gibb, 2005; Tzingounis and Wadiche, 2007; Figure 1). High-affinity NMDARs and mGluRs, located on peri- or extrasynaptic sites (Baude et al., 1993; Paoletti et al., 2013), or on neighboring neurons, mediate most of the glutamatergic spillover responses and their activation is limited by active astrocytic glutamate uptake (Kullmann and Asztely, 1998; Diamond and Jahr, 2000; Huang and Bergles, 2004; Coddington et al., 2013). Therefore, inhibition of EAAT2 favors synaptic crosstalk by the recruitment of neighboring neurons (Huang et al., 2004), or *via* the loss of specificity of afferent inputs (Arnth-Jensen et al., 2002; Scimemi et al., 2004).

EAAT2 blockade results in heterosynaptic LTP in the lateral amygdala (Tsvetkov et al., 2004). LTP at both cortical and thalamic synapses onto pyramidal cells in the lateral amygdala depends on NMDAR activation (Huang and Kandel, 1998; Bauer et al., 2002; Tsvetkov et al., 2002, 2004). Specific pharmacological blockade of EAAT2 with DHK leads to the loss of input specificity in the lateral amygdala, most likely by promoting intersynaptic crosstalk between cortical and thalamic inputs onto pyramidal cells (Tsvetkov et al., 2004). Glutamate spillover may also recruit neighboring interneurons and their activation can impact long-term plasticity expression of principal cells. Indeed, specific blockade of EAAT2 with DHK results in an increased inhibitory drive from GABAergic microcircuits in the striatum (Valtcheva and Venance, 2016). Suprathreshold activation of GABAergic interneurons by cortical stimulation masks the expression of non-timing-dependent LTP of striatal output neurons. In these conditions, enhanced inhibitory transmission promotes the expression of GABA-dependent LTD, instead of postsynaptic NMDAR-mediated LTP, at a narrow temporal window. As a result, postsynaptic NMDAR-mediated LTP is expressed only at a larger temporal window, when the cortical stimulation occurs far from the postsynaptic spike and thus the strong inhibitory drive does not interfere with postsynaptic LTP expression (Valtcheva and Venance, 2016; Figures 2C, 3C).

### EAATs Set the Balance Between LTP and LTD

Interestingly, in many cases of rate-based synaptic plasticity EAATs downregulation by either genetic or pharmacological manipulations leads to a reduction of LTP magnitude or occludes its expression (Katagiri et al., 2001; Wang et al., 2006; Scimemi et al., 2009) and promotes LTD (Brasnjo and Otis, 2001; Massey et al., 2004; Wong et al., 2007; Figure 3A). Therefore, blockade of glutamate transport may alter the balance between LTP and LTD (Katagiri et al., 2001; Scimemi et al., 2009) by reducing LTP magnitude and promoting LTD. Imposing a bias towards synaptic depression in conditions when EAAT activity is downregulated, may result from a shift in the modification threshold for plasticity induction (Cooper and Bear, 2012) by lowering LTD threshold and increasing LTP threshold (Figure 3A). When synaptic glutamate spillover is chronically enhanced and levels of ambient glutamate are thus increased, LTP induction can also be damped by plasticity saturation (Katagiri et al., 2001; Scimemi et al., 2009). In contrast, promoting glutamate spillover can facilitate glutamate access to extrasynaptic glutamate receptors and thus lower LTD threshold (Massey et al., 2004; Wong et al., 2007). It is worthy of note that increasing the frequency of afferent stimulation in control conditions can induce LTD (Figure 3), thus mimicking the effects of EAATs inhibition (Massey et al., 2004). A subtle balance between LTP and LTD should be of crucial importance for optimal memory storage in neural networks and altering glutamate uptake can have profound consequences for learning and memory.

In contrast to the effects triggered by inhibition of glutamate uptake, EAAT2 overexpression with ceftriaxone tends to suppress plasticity expression by altering both LTP and LTD (Omrani et al., 2009; Valtcheva and Venance, 2016; Figures 3B,D). It would be appealing to explore if increasing the frequency of afferent activation could overcome enhanced glutamate uptake by promoting an increased glutamate spillover and, therefore, restoring plasticity.

## Consequences of EAATs Alteration on Behavior

Because EAAT activity controls synaptic plasticity, glutamate uptake is expected to play an essential role in learning and shaping behavior. Although making a causal direct link between molecular impairment with behavioral changes is challenging, we review in this chapter studies correlating the effect of EAATs alterations to behavioral outputs.

*In vivo* chronic blockade of EAAT2 by DHK infusion in the central nucleus of the amygdala induces anxiety (John et al., 2015). This anxiogenic effect could arise from disrupted input specificity in amygdala networks, processing both fear and reward cues, due to enhanced glutamate spillover (Janak and Tye, 2015). Similarly, specific blockade of EAAT2 leads to the loss of specificity of cortical and thalamic inputs onto pyramidal cells in the lateral amygdala, resulting in heterosynaptic LTP of the unpaired input (cortical or thalamic; Tsvetkov et al., 2004). Fear conditioning has been shown to strengthen cortical inputs to the lateral amygdala (Tsvetkov et al., 2002). Therefore heterosynaptic LTP of these inputs following EAAT2 downregulation might impair fear memory formation. The proper functioning of other types of glutamate transporters might also be involved in fear memory formation as mice lacking EAAT3 exhibit decreased freezing behavior following fear conditioning (Wang et al., 2014).

EAATs impairment has consequences on social interactions, reward processing and motivation. EAAT1 knock-out mice exhibit poor nesting behavior and decreased sociability measured by the time spent sniffing an unfamiliar mouse during free social interaction (Karlsson et al., 2009). EAATs blockade in the basolateral amygdala decreases social interactions (Lee et al., 2007). In addition, blocking EAAT2 by *in vivo* DHK infusion in the ventral tegmental area, decreases response to electrical self-stimulation in the medial forebrain bundle (Herberg and Rose, 1990), suggesting that astrocytic glutamate uptake also controls brain reward circuitry. Astrocytic ablation by the gliotoxin L-alpha-aminoadipic acid in the prelimbic part of the prefrontal cortex results in both decreased motivation and increased anxiety (Banasr and Duman, 2008). It is important to note, however, that bulk ablation of astrocytes results not only in decreased levels of astrocytic EAAT2 but it can also impair other astrocytic functions such as gliotransmission and metabolic support, and may result in structural changes of synapses. *In vivo* EAAT2 blockade by DHK in the infralimbic part of the prefrontal cortex (John et al., 2012) or intracerebroventricular infusion of DHK (Bechtholt-Gompf et al., 2010) results in depressive-like behaviors and anhedonia, which are translated by a reduction in motivation and reward-seeking. Interestingly, these effects are prevented by EAAT2 up-regulation following ceftriaxone treatment (Mineur et al., 2007).

In contrast, *in vivo* infusion of DHK in the infralimbic part of the prefrontal cortex reduced despair and anxiety (Gasull-Camós et al., 2017). These effects were not observed when DHK was infused in the prelimbic part of the prefrontal cortex. The effect of increased glutamatergic tone, following DHK infusion, might either stimulate local serotoninergic release or increase the transmission of prefrontal inputs to brainstem serotoninergic neurons and subsequently trigger serotonin release in the infralimbic cortex, thus suppressing anxiety. The molecular pathways and synaptic plasticity mechanisms underlying these phenotypes remain to be determined.

The effects of EAAT2 overexpression on hippocampal learning are complex. EAAT2 overexpression by ceftriaxone impairs LTD and reduces LTP magnitude at the mossy fiber-CA3 synapses (Omrani et al., 2009). Ceftriaxone-treated rats or mice display impaired novel object recognition (Matos-Ocasio et al., 2014; Tian et al., 2019), whereas ceftriaxone administration in mice has no effect on spatial memory (Karaman et al., 2013). Inhibition of EAATs with TBOA facilitates hippocampal LTD induction *in vivo* and *ex vivo*, and disrupts spatial memory retrieval in mice (Wong et al., 2007). CA1 hippocampal LTP is also impaired in knock-out mice for either EAAT2 or EAAT3 (Katagiri et al., 2001; Scimemi et al., 2009), although spatial orientation in the Morris water maze when tested in EAAT3-deficient mice remains unaffected (Peghini et al., 1997).

*In vivo* intrathecal administration of the specific EAAT2 inhibitor DHK impairs LTP of evoked field potentials at C-fiber in the spinal dorsal horn, induced by tetanic stimulation of the sciatic nerve (Wang et al., 2006), and induces spontaneous nociceptive behaviors (Liaw et al., 2005). In addition, an intrathecal infusion of the broad-band EAAT inhibitor TBOA leads to hypersensitivity in response to thermal and mechanical stimuli (Liaw et al., 2005).

Variations in glutamate uptake also affect locomotion. EAAT3 deficient mice display normal motor coordination assessed by rotarod task but a decreased spontaneous locomotion in an open field (Peghini et al., 1997). In contrast, an increased spontaneous locomotion is observed in ceftriaxone-treated rats (Bellesi et al., 2012). Knock-out mice for EAAT1 exhibit motor discoordination in rotarod task (Watase et al., 1998), novelty-induced locomotor hyperactivity measured as an increase in distance traveled during exposure to an open field, and impaired sensory-motor gating translated by reduced acoustic startle response (Karlsson et al., 2008, 2009).

Finally, an inducible astrocytic EAAT2 deletion leads to pathological repetitive behaviors and glutamatergic overactivity (used as a proxy for LTP) in the dorsal striatum (Aida et al., 2015). This is the first study aiming at linking behavior with the effect of EAAT2 downregulation on synaptic plasticity. This spurious plasticity in EAAT2 knock-out mice is likely induced by excessive glutamate spillover as it is dependent on extrasynaptic NMDARs activation. Moreover, the repetitive behavior was diminished by memantine, an extrasynaptic NMDAR antagonist (Aida et al., 2015).

## Physiological Variation of EAATs Levels

### Variations in Glial Coverage and EAATs Expression Across Synapses

Perisynaptic astrocytic processes expressing EAAT1 and EAAT2 are found in all brain regions but the degree of astrocytic coverage is region-specific and the proportion of synapses surrounded by processes can be highly variable within the same structure (Bernardinelli et al., 2014a; Medvedev et al., 2014; Heller and Rusakov, 2015; Khakh and Sofroniew, 2015; Gavrilov et al., 2018). Astrocytic enwrapment of synapses controls the spatial distribution of EAATs and thus their efficiency to uptake glutamate (Oliet et al., 2001; Boudaba et al., 2003; Genoud et al., 2006).

An example of non-uniform distribution of perisynaptic astrocytic processes within the same structure is found in the cerebellum and hippocampus. Climbing fiber–Purkinje cell synapse is extensively enwrapped by astrocytic processes, whereas mossy fiber-granule cell layer synapses (mainly Golgi cells) in the cerebellar vermis are moderately surrounded by astrocytic processes (Xu-Friedman et al., 2001; Xu-Friedman and Regehr, 2003). Mossy fiber-granule cell layer synapses display both rate-based plasticity and STDP depending on the input frequency (D’Errico et al., 2009; Sgritta et al., 2017). Rate-dependent LTD and LTP are induced with LFS (≤1 Hz) and HFS (>50 Hz), respectively, while the frequency range for STDP induction is restricted at the mid-frequency range (6–10 Hz; D’Errico et al., 2009; Sgritta et al., 2017). In the hippocampus, CA3 synapses have lower glial coverage compared with CA1 synapses (Derouiche and Frotscher, 1991; Rollenhagen et al., 2007). Interestingly, upregulation of EAAT2 by ceftriaxone prevents LFS-LTP at mossy fiber-CA3 synapses but not at Schaffer collateral-CA1 (Omrani et al., 2009). LTP at CA3 synapses is mediated by presynaptic kainate receptors (Schmitz et al., 2003), which are particularly sensitive to synaptic glutamate levels (Min et al., 1998), whereas LTP at Schaffer collateral-CA1 depends on postsynaptic NMDARs (Nicoll and Malenka, 1995). Therefore, increasing glutamate uptake *via* EAAT2 upregulation selectively alters HFS-LTP at mossy fiber-CA3 synapses (Omrani et al., 2009).

EAAT density can also differ across similar synapses within the same structure. Purkinje cells in the flocculus express higher levels of EAAT4 than Purkinje cells in the vermis and the regional differences in neuronal transporter density affect the expression of parallel fiber-Purkinje cell LFS-LTD (Wadiche and Jahr, 2005). Interestingly, the rules for induction of Purkinje cell LTD also differ between these two regions. In the vermis, both LFS of parallel fibers and input-timing-dependent stimulation of parallel and climbing fibers result in LFS-LTD and t-LTD, respectively (Wadiche and Jahr, 2005; Safo and Regehr, 2008; Suvrathan et al., 2016). In contrast, Purkinje cells of the flocculus exhibit t-LTD but not LFS-LTD (Wadiche and Jahr, 2005; Suvrathan et al., 2016), suggesting that high EAATs levels might disfavor the expression of rate-based plasticity. Lower EAAT4 expression in the vermis may allow both rate-based and spike-timing-based LTD at Purkinje cells, whereas higher levels of EAAT4 in the flocculus may prevent LFS-LTD, but allow t-LTD to occur at larger temporal intervals (Δt_STDP_ = 120 ms; Suvrathan et al., 2016). This may favor the association of non-coincident inputs occurring with a longer delay than the decay time of transporter currents resulting from parallel fiber stimulation (Wadiche and Jahr, 2005; Suvrathan et al., 2016).

### Plasticity of Glial Coverage and EAATs Expression

Astrocytic enwrapment of neurons controlling glutamate clearance exhibits structural plasticity in response to neuronal activity and can be modulated by experience. The density of astrocytic processes is increased in the layer II/III of the visual cortex of rats which were reared in an enriched environment with new toys and increased social interactions (Jones et al., 1996). Another type of sensory experience such as prolonged whisker stimulation increases the astrocytic enwrapment of synapses and EAAT2 expression in the sensory cortex (Genoud et al., 2006). Interestingly, following whisker stimulation, the motility of astrocytic endfeets is increased specifically in the corresponding whisker barrel while stimulation of the surrounding whisker fails to induce such effect (Bernardinelli et al., 2014b). Negative experience like stress alters hippocampal EAAT2 expression with a biphasic profile: acute stress, *via* tail shock, downregulates EAAT2 levels (Yang et al., 2005), whereas chronic restraint stress upregulates EAAT2 (Reagan et al., 2004). Different learning paradigms can also trigger structural plasticity of astrocytes and EAATs expression. Motor-skill learning induces glial hypertrophy in the molecular layer of cerebellum leading to a greater volume of glia per Purkinje cell, possibly limiting glutamate spillover (Anderson et al., 1994). Similarly, voluntary free exercise on a running wheel for 3 weeks results in increased ramification and more complex morphology of astrocytic processes in the globus pallidus of mice. These changes are correlated with sustained physical exercise since they disappear after a resting period of additional 3 weeks (Tatsumi et al., 2016). Contextual fear conditioning increases the rate of glutamate uptake and EAAT3 membrane expression in the hippocampal CA1 region (Levenson et al., 2002). This suggests that increased glial ensheathment of synapses and upregulation of glutamate uptake may be especially important for counterbalancing the increased synaptic efficacy and maintaining the synaptic strength following experience-dependent potentiation. Indeed, hippocampal LTP is associated with an increase in the astrocytic coverage of pre- and postsynaptic elements (Lushnikova et al., 2009), an increase in the EAAT3- and EAAT2-dependent glutamate uptake during the early and late phase of LTP, respectively, and EAAT3 translocation from the cytosol to the plasma membrane (Diamond et al., 1998; Lüscher et al., 1998; Levenson et al., 2002; Kawamura et al., 2004; Pita-Almenar et al., 2005, 2006). In addition, LTP-induced astrocytic group-I mGluR-dependent potentiation of EAAT2 glutamate uptake, as well as membrane insertion of EAAT1, has been reported (Shen and Linden, 2005; Devaraju et al., 2013).

Decrease in the glial enwrapment of synapses onto oxytocinergic neurons in the hypothalamus of female rats occurs with the transition to motherhood during the lactation period and these changes are reversed in post-lactating animals (Theodosis and Poulain, 1993). This results in an increase in the tonic glutamate concentration likely due to altered glutamate clearance (Oliet et al., 2001). This, in turn, decreases the probability of glutamate release by activation of presynaptic type-III mGluRs mediated by glutamate spillover (Oliet et al., 2001). Similar retraction of astrocytic endfeet occurs with chronic dehydration when rats drink hypertonic saline for several days (Perlmutter et al., 1985; Chapman et al., 1986), which results in a decreased expression of EAAT2 and activation of presynaptic type-III mGluRs (Boudaba et al., 2003). Increased tonic glutamate concentration activates postsynaptic GluN2B-NMDARs and increases neuronal excitability and firing frequency of hypothalamic neurons (Fleming et al., 2011; Naskar and Stern, 2014). Interestingly, both HFS-LTP and LFS-LTD induced in the hypothalamus of female rats are NMDAR-dependent (Panatier et al., 2006a) and, therefore, might be sensitive to changes in glutamate uptake triggered by astrocytic structural plasticity. The switch in the threshold for plasticity induction in lactating rats is attributed to a decreased GluN2B-NMDARs activation caused by a deficiency in D-serine signaling (Panatier et al., 2006b). An alternative mechanism could be that the retraction of astrocytes and increased glutamate uptake may serve as a high-pass filter, increasing HFS-LTP threshold by prioritizing high-frequency afferent inputs.

Physiological fluctuations in the glial coverage of neurons can occur in a cyclic manner. Indeed, in the arcuate hypothalamic nucleus, the density of astrocyte cell bodies and processes revealed an increased on the afternoon of proestrus and on the morning of estrus compared to other phases of the oestrous cycle. This structural plasticity is dependent on estradiol and absent in ovariectomized rats (Garcia-Segura et al., 1994). Neurons in the suprachiasmatic hypothalamic nucleus undergo rhythmic ultrastructural rearrangements in their astrocytic coverage over the circadian cycle (Lavialle et al., 2001; Becquet et al., 2008). Importantly, this structural plasticity is synapse-specific. At nighttime, the glial coverage of neurons expressing the vasoactive intestinal peptide increases, whereas the glial coverage of arginine vasopressin-expressing neurons decreases (Becquet et al., 2008). Glutamate uptake is crucial in the regulation of neuronal circadian oscillations (Brancaccio et al., 2017). Sleep deprivation induces upregulation of EAAT1 and increased glial coverage in the prefrontal cortex (Bellesi et al., 2015). In contrast, sleep deprivation has a synapse-specific effect in the lateral hypothalamus as it decreases EAAT2 expression around wake-promoting orexin neurons, while it increases EAAT2 around sleep-promoting melanin-concentrating hormone neurons (Briggs et al., 2018). In conclusion, EAATs exert a tight control of endogenous rhythms by regulating the degree of coordination between neurons and most likely by setting the threshold for synaptic plasticity induction.

It should be taken into consideration that some experimental conditions can affect glial coverage and thus synaptic plasticity. Indeed, acute brain slice preparation and incubation methods (Table 1) may alter the ultrastructure of the neuropil (Bourne et al., 2007; Bourne and Harris, 2012; Harris and Bourne, 2012) and likely the morphology of the fine astrocytic processes, which in turn could affect long-term synaptic plasticity expression. Exposure to ice-cold artificial cerebrospinal fluid (ACSF) has been reported to induce synaptogenesis and increase in spine density in acute hippocampal slices, which can be prevented if slices are prepared at room temperature (Bourne et al., 2007; Bourne and Harris, 2012). Similarly, incubation of acute hippocampal slices in submerged chamber results in a loss or retraction of astrocytic process but can be prevented if slices are incubated in an interface chamber (Harris and Bourne, 2012).

## Conclusions and Future Directions

Here, we reviewed studies evidencing that the subtle equilibrium between the localization and density of EAATs, together with the glial coverage of neurons, shapes not only excitatory transmission but also long-term synaptic plasticity, thus impacting on learning and memory. Interestingly, baseline GABAergic is not affected by the decrease in the astrocytic enwrapment of neurons and increased levels of ambient glutamate. Indeed, resting glutamate concentrations are not sufficient to tonically activate type-III mGluRs located on GABAergic terminals in the hypothalamus of lactating rats (Piet et al., 2004). However, the evoked inhibitory transmission is decreased when glutamatergic terminals are activated. This results in intersynaptic crosstalk and activation of presynaptic type-III mGluRs (Piet et al., 2004), or kainate receptors (Bonfardin et al., 2010) on GABAergic terminals (Figure 1). It remains to determine whether, in addition to glutamatergic synaptic plasticity (Figure 3), EAATs also control long-term synaptic plasticity of GABAergic inputs. Physiological fluctuations (Theodosis and Poulain, 1993; Garcia-Segura et al., 1994; Becquet et al., 2008; Briggs et al., 2018) and regional differences (Wadiche and Jahr, 2005) in the glial coverage of neurons and EAATs levels might thus regulate the balance between synaptic excitation and inhibition and shape excitatory and inhibitory synaptic plasticity (Vogels et al., 2013).

Increase in the glial coverage of neurons together with EAATs expression and/or proximity to presynaptic release sites would limit glutamate access to receptors involved in synaptic plasticity (Omrani et al., 2009; Valtcheva and Venance, 2016), thus increasing the threshold for plasticity induction and preventing synaptic crosstalk. Indeed, protrusion of astrocytic processes increases EAAT2 activity, without altering EAAT2 expression, and decreases the magnitude of HFS-LTP at Schaffer collateral-CA synapses (Pannasch et al., 2014). EAAT2 might differentially control synaptic plasticity depending on the type of postsynaptic neuron. Interestingly, synaptic glutamatergic tone is higher around inhibitory neurons compared to excitatory neurons in the prefrontal cortex likely caused by a difference in the density or activity of EAAT2 (Yao et al., 2018). Therefore long-term synaptic plasticity of inhibitory neurons (Kullmann and Lamsa, 2007; Fino and Venance, 2011) might be particularly sensitive to upregulation of glutamate uptake.

Under weak glial coverage of neurons (Perlmutter et al., 1985; Theodosis and Poulain, 1993; Becquet et al., 2008), the ambient glutamatergic tone is high due to decreased glutamate uptake (Oliet et al., 2001). This may cause synapses to become less sensitive to the precise timing of pre- and post-synaptic spikes, thus inducing a switch from spike-timing- to rate-coding. In this scenario, only high-frequency trains of presynaptic activity would be successful in inducing long-term synaptic plasticity. EAATs expression and their proximity to the synapse may, therefore, serve as a metaplastic mechanism to influence activity-dependent plasticity induction. Adjusting neuronal computations by EAATs may be important for synapses to adapt to different physiological demands (during sleep/wake cycle, circadian cycle, estrous cycle, lactation).

The question of whether individual neurons encode and process information by using precise spike timings, thus, working as coincidence detectors, or spike rates, thus, working as temporal integrators, has been debated (deCharms and Zador, 2000). Both mechanisms generally coexist in the same neuron. In the prefrontal cortex, depending on the synaptic inputs to layer V pyramidal neurons, dendrites behave either as temporal integrators or as coincident detectors (Dembrow et al., 2015). Neurons in the subthalamic nucleus operate by combining integration and coincidence detection depending on the ongoing presynaptic activity (Farries et al., 2010). Theoretical work has shown that cortical pyramidal neurons are capable of operating in a continuum between temporal integration and coincidence detection, depending on the characteristics of the synaptic inputs (Rudolph and Destexhe, 2003). Here, we hypothesize that EAATs levels, their distribution and/or astrocytic enwrapment of neurons may define if neurons operate in a rate- or spike-timing-based manner.

Future advances in *in vivo* two-photon imaging would allow chronic (hours or days) monitoring of astrocytic processes across different physiological states. This will make possible to image the motility of perisynaptic astrocytic endfeets around fluorescently-tagged spines of identified neuronal populations. Simultaneous monitoring of astrocytic structural rearrangements and dendritic spine remodeling of specific neuronal types will help establish a model of their dynamic relationship during learning, sensory stimulation and physiological rhythms. Perisynaptic astrocytic endfeets are highly dynamic *in vivo* (Bernardinelli et al., 2014b), and coupling *in vivo* imaging of astrocytes with *in vivo* whole-cell recordings of individual neurons would allow correlating astrocytic structural rearrangement and synaptic transmission and plasticity.

Genetic and pharmacological approaches have proved the crucial involvement of EAATs in synaptic plasticity. However, these tools do not provide information about the dynamics of EAATs activity at fast timescale, which may be essential for regulating the activation of synaptic and extrasynaptic glutamate receptors and thus shaping different forms of plasticity. With the rise of new tools allowing photocontrol of protein activity (Kramer et al., 2013; Durand-de Cuttoli et al., 2018) and high-speed glutamate imaging *via* the intensity-based glutamate-sensing fluorescent reporter iGluSnFR and its fast variants (Marvin et al., 2013; Parsons et al., 2016; Helassa et al., 2018), it would be possible to manipulate EAATs activity at a millisecond timescale and monitor the time course of glutamate in coordination with pre- and postsynaptic activity. An important advance of optical methods compared to biochemical assays in measuring glutamate transport is that the time course of evoked fluorescent responses reflects transporter-mediated uptake as well as glutamate diffusion. Another crucial factor is that genetically encoded sensors can be driven under the control of a specific promoter allowing measurement of glutamate sensed at the plasma membrane of neurons, compared to glutamate responses at the astrocytic membrane measured by astrocytic transporter currents. With these powerful tools, it would be particularly interesting to explore whether EAATs activity and astrocytic coverage of synapses across different brain regions tune the sensitivity of synapses to rate- and/or STDP paradigms (Wadiche and Jahr, 2005; Suvrathan et al., 2016).

## Author Contributions

SV and LV wrote the review article, have edited and corrected the manuscript. SV did the figures and the table.

## Conflict of Interest Statement

The authors declare that the research was conducted in the absence of any commercial or financial relationships that could be construed as a potential conflict of interest.
